# Draft genome sequence data of the anaerobic, thermophilic, chitinolytic bacterium strain UUS1-1 belonging to genus *Hydrogenispora* of the uncultured taxonomic OPB54 cluster

**DOI:** 10.1016/j.dib.2020.106528

**Published:** 2020-11-14

**Authors:** Umbhorn Ungkulpasvich, Ayaka Uke, Sirilak Baramee, Akihiko Kosugi

**Affiliations:** a Graduate School of Life and Environmental Sciences, University of Tsukuba, 1-1-1 Tennodai, Tsukuba, Ibaraki 305-8572, Japan; b Biological Resources and Post-Harvest Division, Japan International Research Center for Agricultural Sciences (JIRCAS), 1-1 Ohwashi, Tsukuba, Ibaraki 305-8686, Japan

**Keywords:** Chitin, Degradation, Thermophilic, OPB54, *Hydrogenispora*, Draft genome

## Abstract

Strain UUS1-1 (=JCM33882 =DSM111537) is a novel chitinolytic, thermophilic, anaerobic bacterium belonging to the genus *Hydrogenispora* of the uncultured taxonomic OPB54 cluster within the phylum Firmicutes. Strain UUS1-1 has a unique, long, hair-like rod morphology and a strong ability to degrade crystalline chitin. The whole genome of strain UUS1-1 was sequenced on an Ion GeneStudio S5 system, which yielded 86 contigs comprising 2,482,547 bp, 2235 protein-coding sequences, and a G+C content of 52.1 mol%. Strain UUS1-1 is the second cultivable isolate, besides *H. ethanolica*, within the OPB54 cluster and may be classified as a novel species. The genomic data have been deposited at the National Center for Biotechnology Information (NCBI) under accession number JAAKDE00000000.

## Specifications Table

**Table:** 

Subject	Microbiology
Specific subject area	Bacteriology, Genomics
Type of data	Table, Figure
How data were acquired	Whole-genome sequencing using the Ion GeneStudio S5 system
Data format	Raw, Analyzed
Parameters for data collection	Genomic DNA was extracted from a pure culture of strain UUS1-1 (JCM 33882), which belongs to the genus *Hydrogenispora*. The genome of strain UUS1-1 was sequenced using the Ion GeneStudio S5 system, assembled *de novo* using the CLC Genomics Workbench 20.0.1, and annotated using the NCBI Prokaryotic Genome Annotation Pipeline.
Description of data collection	Genomic DNA was extracted from strain UUS1-1. Following whole-genome sequencing, the genome was assembled and annotated.
Data source location	Japan International Research Center for Agricultural Sciences (JIRCAS), Tsukuba, Ibaraki, Japan
Data accessibility	Repository name: NCBI Data identification number: JAAKDE000000000. The version described in this paper is JAAKDE010000000.1 Direct URL to data: https://www.ncbi.nlm.nih.gov/nuccore/JAAKDE000000000.1 The BioProject ID in GenBank is PRJNA607398 (https://www.ncbi.nlm.nih.gov/bioproject/PRJNA607398) The BioSample ID in GenBank is SAMN13885186 (https://www.ncbi.nlm.nih.gov/biosample/SAMN13885186)

## Value of the Data

• Strain UUS1-1 is a novel, anaerobic, thermophilic, chitinolytic bacterium belonging to the genus *Hydrogenispora* of the uncultured taxonomic OPB54 cluster.
• The data from this draft genome can help the scientific community understand the genetic properties of this unique bacterial taxon which currently contains only one other cultivated isolate (*Hydrogenispora ethanolica*). In addition, these data will be valuable to engineering researchers that study thermophilic and chitinolytic enzymes.
• The data from this draft genome provide new insights into crystalline chitin degradation ability in thermophilic conditions and properties of the genus *Hydrogenispora*.

## 1 Data Description

Chitin is an insoluble linear chain of β-1,4-*N*-acetylglucosamine (GlcNAc). This crystalline polymer is the major component of the exoskeletons of arthropods, such as shrimps and insects [1, 2]. Chitin and its derivatives can be transformed into highly valuable compounds that have many industrial applications. Chitinase-producing microorganisms have been isolated from multiple environments. Bacterial chitinases hold promise for use in several commercial applications, such as production of GlcNAc and chitosan. Thermostable chitinases from bacteria may have numerous applications because such chitinases would likely reduce industrial costs [3, 4]; however, the chitinases currently used for industrial applications are mainly from mesophilic bacteria [5]. Thus, screening, identifying, and studying the functional properties of thermophilic chitinases is crucial. Currently, there is limited information describing the participation of anaerobic thermophilic bacteria in insoluble chitin degradation systems.

Strain UUS1-1, classified as belonging to the genus *Hydrogenispora* and the uncultured taxonomic OPB54 cluster, was successfully isolated as a pure culture from a bacterial community. Strain UUS1-1, deposited at the RIKEN BioResource Research Center as JCM33882 and the German Collection of Microorganisms and Cell Cultures GmbH (DSMZ) as DSM111537, possesses unique features such as a hair-like rod morphology, and high chitinase activity in thermophilic conditions. Strain UUS1-1 is the first anaerobic, thermophilic, chitinolytic bacterium, and the second cultivated isolate besides *H. ethanolica* that belongs to the OPB54 cluster [6].

We sequenced the genome of strain UUS1-1 to obtain new information on anaerobic, thermophilic chitin degradation systems within the genus *Hydrogenispora* and the taxonomic cluster OPB54, which belongs to an unidentified taxon at the order- or class-level. Features of the genome are shown in Table 1. DNA sequencing, performed using the Ion GeneStudio S5 system, generated 11,760,377 single reads with an average length of 187 bp. The genome was assembled *de novo* using CLC Genomics Workbench 20.0.1 (CLC Bio, Qiagen, Valencia, CA), which resulted in 86 contigs with an N50 of 117,588 bp and a maximum size of 238,885 bp. The genome of strain UUS1-1 comprised 2,482,547 bp and had a G+C content of 52.1 mol%. Interestingly, the genome of the most closely related strain in this genus, *H. ethanolica* strain LX-B, was nearly identical in its G+C content but 2.4 times the size (5,983,461 bp, G+C content 54.2 mol%).

**Table 1:** Features of the strain UUS1-1 genome.

Feature	Description
Number of reads used in the assembly	11,760,377
Mean read length	187 bp
Genome size	2,482,547 bp
Number of contigs	86
N50 contig length	117,588 bp
Mean contig length	28,867 bp
Genome coverage depth	1,469-fold
G+C content	52.1 mol%
Total number of genes	2,337
Number of CDSs	2,235
Number of rRNAs	10
Number of tRNAs	48
Number of CRISPRs	4

CDS, coding sequence. CRISPRs, clustered regularly interspaced short palindromic repeats.

**Fig. 1:**
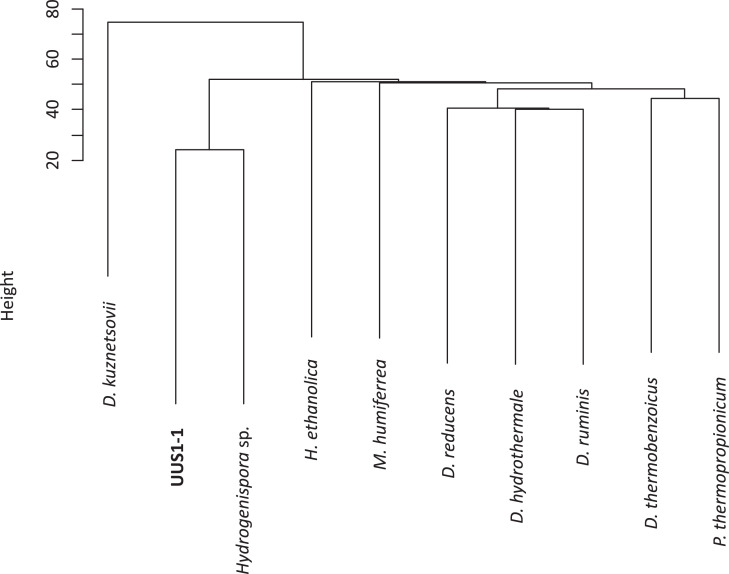
Dendrogram of average nucleotide identity (ANI) values. The ANI value of each pair of strains was calculated, and a dendrogram was constructed using the unweighted pair group method with arithmetic means. The strains which were used for this calculation and the dendrogram were: UUS1-1, *Desulfofundulus kuznetsovii* (NZ_LGGU01000000), *Desulfofundulus thermobenzoicus* (NZ_WHYR01000000), *Desulfotomaculum hydrothermale* (NZ_FQXF01000000), *Desulfotomaculum reducens* (NC_009253), *Desulfotomaculum ruminis* (NC_015589), *Hydrogenispora ethanolica* (NZ_SLUN01000000), *Hydrogenispora* sp. (DUQQ01000000), *Pelotomaculum thermopropionicum* (AP009389), and *Moorella humiferrea* (PVXM00000000)*.*

Using a phylogenetic tree based on 16S rRNA gene sequences, we determined that *H. ethanolica* strain LX-B (90.4% similarity, accession no. SLUN00000000) is the only other member of the uncultured taxonomic OPB54 cluster (phylum Firmicutes) [6]. However, the mesophilic anaerobic bacterium *H. ethanolica* has very different phenotypic characteristics to strain UUS1-1 (GenBank accession number of 16S rRNA sequence: MN602556). We calculated the average nucleotide identity (ANI) [7] of strain UUS1-1 and nine strains from the genera *Hydrogenispora, Desulfofundulus, Desulfotomaculum, Pelotomaculum*, and *Moorella.* This analysis showed a 65.5% ANI between strain UUS1-1 and *H. ethanolica* strain LX-B, and a 82.9% ANI between strain UUS1-1 and a *Hydrogenispora* sp. isolate (DUQQ01000000) that was identified by comprehensive genome-resolved metagenomics [8] (Fig. 1, Suppl. Tables 1 and 2). The ANI was <65% for all other strains included in the analysis (Suppl. Tables 1 and 2). These results indicate that strain UUS1-1 represents a novel species.

Genome annotation was carried out via the NCBI Prokaryotic Genome Annotation Pipeline (PGAP) [9]. Strain UUS1-1 possessed 2,337 total predicted genes; 2,235 protein-coding sequences (CDSs); 4 rRNA genes (encoding two 5S rRNAs, one 16S rRNA, and one 23S rRNA); 47 tRNA genes; and 4 clustered regularly interspaced short palindromic repeats. The predicted CDSs were assigned putative functions using the 25 Clusters of Orthologous Groups. The presence of predicted enzymes associated with chitin metabolism in strain UUS1-1 was confirmed for chitinase (EC 3.2.1.14), *N*-acetylglucosaminidase (EC 3.2.1.52), chitin deacetylase (EC 3.1.1.72/ EC 3.5.1.41), glucosidase (EC 3.2.1.20/ EC 3.2.1.21), glucokinase (EC 2.7.1.2), glucosamine-fructose-6-phosphate aminotransferase (EC 2.6.1.16), phosphoglucosamine mutase (EC 5.4.2.10), glucosamine-6-phosphate deaminase (EC 3.5.99.6), and glucosamine-1-phosphate *N*-acetyltransferase/ UDP-*N*-acetylglucosamine pyrophosphorylase (EC 2.3.1.157/ EC 2.7.7.23) (Table 2). The predicted chitinases and chitin deacetylases may play an important role in the efficient degradation of crystalline chitin by strain UUS1-1. The genome of strain UUS1-1 will be useful in future studies that characterize other bacteria belonging to the OPB54 cluster, and it will inform new applications for chitin usage technologies in thermophilic conditions (https://www.ncbi.nlm.nih.gov/nuccore/JAAKDE000000000.1).

**Table 2:** Predicted genes encoding chitinolytic and related enzymes.

Function	EC #	Predicted gene(s)
Chitinase	EC 3.2.1.14	WP_181339859.1, WP_181339858.1, WP_181339894.1, WP_181340572.1, WP_181340429.1
*N*-acetylglucosaminidase	EC 3.2.1.52	WP_181339652.1
Chitin deacetylase	EC 3.1.1.72/ EC 3.5.1.41	WP_181339645.1, WP_181340147.1, WP_181340352.1, WP_181339341.1, WP_181339805.1, WP_181339807.1, WP_181339869.1
Glucosidase	EC 3.2.1.20/ EC 3.2.1.21	WP_181340416.1, WP_181338805.1
Glucokinase	EC 2.7.1.2	WP_181340205.1, WP_181339717.1
Glucosamine-fructose-6-phosphate aminotransferase	EC 2.6.1.16	WP_013809510.1
Phosphoglucosamine mutase	EC 5.4.2.10	WP_007289355.1
Glucosamine-6-phosphate deaminase	EC 3.5.99.6	WP_013298094.1
Glucosamine-1-phosphate *N*-acetyltransferase/ UDP-*N*-acetylglucosamine pyrophosphorylase	EC 2.3.1.157/ EC 2.7.7.23	WP_007506043.1

## 2 Experimental Design, Materials and Methods

### 2.1 Genomic DNA extraction and sequencing

Genomic DNA of strain UUS1-1 was prepared by phenol/chloroform extraction from cells grown at 60 °C with glucose as the carbon source [10]. Fragmentation and library preparation of DNA was performed using an Ion Xpress Plus Fragment Library Kit (Thermo Fisher Scientific, Waltham, MA, USA) according to the manufacturer’s protocol, which generated fragments with an average length of 400 bp. Fragments of approximately 200 to 300 bp were size-selected by electrophoresis on E-Gel SizeSelect II agarose gels (Invitrogen, Thermo Fisher Scientific, Waltham, MA, USA) before library preparation. The genomic DNA sequences of strain UUS1-1 were obtained using an Ion GeneStudio S5 system and processed.

### 2.2 Genome assembly and annotation

*De novo* genome assembly was performed using CLC Genomics Workbench version 20.0.1. after removal of low-quality reads. The genome was annotated using the NCBI PGAP [9]. The prediction of function of each annotated gene was executed within the Integrated Microbial Genomes-Expert Review system developed by the Joint Genome Institute (Walnut Creek, CA, USA) [11].

### 2.3 Genomic ANI

Pairwise ANI values from the whole-genome sequences of two *Hydrogenispora* strains, two *Desulfofundulus* strains, three *Desulfotomaculum* strains, one *Pelotomaculum* strain, and one *Moorella* strain were calculated using GENETYX NGS version 4.1.1 with the BLASTALL algorithm. The matrix generated from the ANI values among these nine strains and strain UUS1-1 was converted into a genetic dendrogram using algorithms such as the unweighted pair group method with arithmetic means and the single-linkage clustering method in the R statistical program [7].

## Declaration of Competing Interest

The authors declare that they have no known competing financial interests or personal relationships which have, or could be perceived to have, influenced the work reported in this article.

## Appendix Supplementary materials

Image, application 1

Image, application 2
