# Supplementary material for: Draft genome sequence data of the anaerobic, thermophilic, chitinolytic bacterium strain UUS1-1 belonging to genus Hydrogenispora of the uncultured taxonomic OPB54 cluster
Source: Data Brief. 2020 Nov 14;33:106528. doi: 10.1016/j.dib.2020.106528 (PMC7708789; doi:10.1016/j.dib.2020.106528)
Supplement: Supplementary file 1 [file mmc1.docx]

Supplementary Table 1. Strains included in the dendrogram based on average nucleotide identity (ANI) values (Figure 1).

| Organism | Accession no. |
| --- | --- |
| Strain UUS1-1 | JAAKDE00000000 |
| *Desulfofundulus kuznetsovii* | NZ_LGGU01000000 |
| *Desulfofundulus thermobenzoicus* | NZ_WHYR01000000 |
| *Desulfotomaculum hydrothermale* | NZ_FQXF01000000 |
| *Desulfotomaculum reducens* | NC_009253 |
| *Desulfotomaculum ruminis* | NC_015589 |
| *Hydrogenispora ethanolica* | NZ_SLUN01000000 |
| *Hydrogenispora* sp. | NZ_ DUQQ01000000 |
| *Pelotomaculum thermopropionicum* | AP009389 |
| *Moorella humiferrea* | PVXM00000000 |

Supplementary Table 2. Pairwise ANI values among strain UUS1-1, *Desulfofundulus* spp., *Desulfotomaculum* spp., *Hydrogenispora* spp., *Pelotomaculum thermopropionicum*, and *Moorella humiferrea*.

|  | UUS1-1 | *D. kuznetsovii* | *D. thermobenzoicus* | *D. hydrothermale* | *D. reducens* | *D. ruminis* | *H. ethanolica* | *Hydrogenispora* sp*.* | *P. thermopropionicum* | *M. humiferrea* |
| --- | --- | --- | --- | --- | --- | --- | --- | --- | --- | --- |
| UUS1-1 | 100 |  |  |  |  |  |  |  |  |  |
| *D. kuznetsovii* | 62.02 | 100 |  |  |  |  |  |  |  |  |
| *D. thermobenzoicus* | 63.78 | 65.65 | 100 |  |  |  |  |  |  |  |
| *D. hydrothermale* | 63.14 | 64.23 | 66.93 | 100 |  |  |  |  |  |  |
| *D. reducens* | 63.84 | 62.79 | 65.18 | 71.25 | 100 |  |  |  |  |  |
| *D. ruminis* | 64.01 | 63.9 | 66.93 | 71.68 | 71.25 | 100 |  |  |  |  |
| *H. ethanolica* | 65.47 | 61.6 | 64.45 | 63.32 | 62.61 | 63.64 | 100 |  |  |  |
| *Hydrogenispora* sp*.* | 82.93 | 61.53 | 63.87 | 62.8 | 62.15 | 62.51 | 65.24 | 100 |  |  |
| *P. thermopropionicum* | 63.06 | 64.67 | 68.74 | 65.9 | 65.62 | 65.78 | 63.43 | 62.87 | 100 |  |
| *M. humiferrea* | 63.4 | 62.62 | 63.97 | 64.29 | 63.99 | 65.19 | 63.67 | 63.55 | 63.63 | 100 |
